# Orbitofrontal Gray-White Interface Injury and the Association of Soccer Heading With Verbal Learning

**DOI:** 10.1001/jamanetworkopen.2025.32461

**Published:** 2025-09-18

**Authors:** Joan Y. Song, Roman Fleysher, Kenny Ye, Mimi Kim, Walter F. Stewart, Molly E. Zimmerman, Richard B. Lipton, Michael L. Lipton

**Affiliations:** 1 Dominick P. Purpura Department of Neuroscience, Albert Einstein College of Medicine, Bronx, New York; 2 Department of Radiology, Columbia University Irving Medical Center, New York, New York; 3 Department of Epidemiology and Population Health, Albert Einstein College of Medicine, Bronx, New York; 4 Medcurio Inc, Oakland, California; 5 Department of Psychology, Fordham University, Bronx, New York; 6 Saul R. Korey Department of Neurology, Albert Einstein College of Medicine and Montefiore Medical Center, Bronx, New York; 7 Department of Biomedical Engineering, Columbia University, New York, New York

## Abstract

**Importance:**

Sport-related repetitive head impacts (RHIs) are linked to structural and functional brain changes and may be associated with risk for neurodegenerative disease. However, the locus of RHI-associated pathology that leads to adverse cognitive performance has yet to be characterized in vivo.

**Objective:**

To examine whether orbitofrontal gray matter–white matter interface (GWI) microstructure is associated with soccer RHI and whether it mediates the association of RHI with cognitive performance.

**Design, Setting, and Participants:**

This cross-sectional study, performed at Albert Einstein College of Medicine and Columbia University Irving Medical Center, included a population-based sample from the greater New York City area of amateur soccer players aged 18 to 55 years who played for at least 5 years, currently played at least 6 months per year, and were fluent in English. Exclusion criteria consisted of schizophrenia, bipolar disorder, current substance use disorder, any drug use within 30 days, and any contraindication to magnetic resonance imaging. Data were collected from November 11, 2013, to December 28, 2015, and analyses were performed from April 1 through December 4, 2024.

**Exposure:**

Soccer RHI exposure in the prior 12 months quantified via the HeadCount 12-month survey.

**Main Outcome and Measures:**

Slope of diffusion magnetic resonance imaging metrics (fractional anisotropy [FA], axial diffusivity, orientation dispersion index, and intracellular volume fraction) across the GWI was computed for each of 6 cerebral regions (cingulate, orbitofrontal, frontal, temporal, parietal, and occipital). The GWI slope association with RHI and with International Shopping List (ISL) immediate recall task performance, which is associated with RHI, were examined. Mediation analyses tested for the mechanistic role of GWI slope in the RHI and ISL association.

**Results:**

In 352 adult amateur soccer players aged 18 to 53 years (243 [69.0%] male; mean [SD] age, 25.6 [7.5] years), greater RHI exposure was associated with a less steep FA slope across the orbitofrontal GWI (estimate, 0.000001; *P* < .001). The orbitofrontal FA slope measure mediated the association of RHI with ISL performance (indirect effect estimate, −0.000064; *P* = .006).

**Conclusions and Relevance:**

In this cross-sectional study of soccer RHI, the orbitofrontal GWI emerged as a specific site of soccer RHI-related consequences, with FA slope suggesting that GWI microstructural disruption plays a mediating role in the association of greater RHI exposure with poorer verbal learning task performance.

## Key Points

**Question:**

Is the orbitofrontal gray matter–white matter interface (GWI) a locus of injury due to repetitive head impacts (RHIs) among soccer players, and does it mediate RHI associations with poorer verbal learning?

**Findings:**

In this cross-sectional study of RHI in 352 adult amateur soccer players, greater RHI exposure was associated with attenuation of orbitofrontal GWI fractional anisotropy slope sharpness, and orbitofrontal fractional anisotropy slope mediated the association between RHI and International Shopping List Task score.

**Meaning:**

These findings suggest that soccer RHI-related consequences are specific to orbitofrontal GWI, where disruption plays a mediating role in the adverse association of RHI with cognitive performance.

## Teaser

This cross-sectional study assesses whether attenuation of the normally sharp microstructural transition at the orbitofrontal gray matter–white matter interface mediates associations of prior 12-month soccer heading and injuries with poorer verbal learning among adult amateur players.

## Introduction

Repetitive head impacts (RHIs) in sport are a growing concern given evidence that repeated low-level impacts, even in the absence of diagnosed concussion, lead to adverse effects and increased risk for neurodegenerative disease.^1^ Soccer is the most popular sport worldwide^2^ and, in part due to the practice of heading (ie, intentionally hitting the ball with the head), may be the greatest source of RHI.^3^ Soccer heading has been associated, in an exposure-dependent manner, with central nervous system symptoms, adverse changes to brain microstructure measured with diffusion magnetic resonance imaging (dMRI), and worse cognitive function.^4,5,6,7,8,9,10,11,12,13,14^ Chronic traumatic encephalopathy (CTE), a neurodegenerative disease associated with RHI, has been identified in both former professional and young adult amateur soccer players.^15,16^ However, some studies have not found a clear link between heading and adverse outcomes, particularly in the acute time frame.^17,18^ For example, Kontos et al^11^ found no conclusive evidence linking heading to adverse outcomes in their meta-analysis of cognitive function, symptoms, and balance. However, they acknowledged that heterogeneity across studies diminished the power of the meta-analysis to identify salient effects. These limitations underscore the need for further research to understand the association of heading with adverse effects.

The cortical gray matter–white matter interface (GWI) is known to be susceptible to trauma, based on biomechanical modeling,^19^ histological evidence in animals^20,21,22,23,24^ and humans,^25^ and MRI findings in animals^26^ and human patients with traumatic brain injury (TBI).^27,28,29,30,31,32^ Specific to RHI, finite element modeling has identified the orbitofrontal region, a well-known injury predilection site in TBI,^33^ as the site of greatest tissue strain from soccer heading.^34^ American football players exhibited postmortem astrogliosis at the GWI despite absence of other pathology, including CTE.^25^ In vivo GWI neuroinflammation has been identified in RHI-exposed military personnel using positron emission tomography.^35^ These studies implicate the GWI as a probable locus of RHI-associated brain pathology.

Despite its potential importance as a site of injury from RHI, the GWI is routinely excluded from dMRI analyses due to potential partial volume effects and misregistration. Prior studies thus leave a critical gap in our understanding of GWI vulnerability. Our group developed a method that exploits the marked difference in diffusion anisotropy between gray matter and white matter at the GWI.^36,37^ Rather than measuring magnitude of diffusion parameters at a discrete region of interest, our group profiled the spatial transition of dMRI microstructural measures across the GWI, recapitulating expected aging effects.

Our group previously reported, among young adult soccer athletes, exposure-dependent associations of RHI with lower fractional anisotropy (FA) slope in deep white matter regions and, in parallel, with lower performance on cognitive tests.^38,39,40^ However, to our knowledge, no study to date has tested the mediating role of dMRI or any imaging measure in the association of RHI with cognitive performance.

This study tested the hypothesis that attenuation of the normally sharp microstructural transition at the orbitofrontal GWI, a location of shear force trauma during RHI, mediates associations of prior 12-month soccer heading with poorer verbal learning among adult amateur players. We quantified the sharpness of the GWI microstructural profile to identify microstructural injury and its mediation of the RHI and verbal learning association.

## Methods

### Study Participants

In this cross-sectional study, we analyzed dMRI and cognitive performance in adult amateur soccer players from the New York City area. The Albert Einstein College of Medicine and Columbia University institutional review boards approved the study. Data were collected from November 11, 2013, to December 28, 2015, and analyses were performed from April 1 through December 4, 2024. Participants were recruited via advertisement and social media, and interested respondents were screened for eligibility. All participants gave written informed consent. This study followed the Strengthening the Reporting of Observational Studies in Epidemiology (STROBE) reporting guideline.

Inclusion criteria were ages 18 to 55 years, amateur soccer play for 5 years or longer, current play at least 6 months per year, and fluency in English. The original study^40^ was designed to assess the effect of prior RHI. Inclusion criteria were specified a priori to identify participants with substantial prior and current active play while ensuring a broad pool of potential participants. Exclusion criteria were schizophrenia, bipolar disorder, known neurological disorders such as dementia and migraine, prior moderate or severe TBI, acute (within 3 months) or persistent symptomatic concussion (mild TBI), current substance use disorder, illicit drug use within 30 days, and contraindication to MRI.^40^ Participants were excluded if they reported illicit drug use (including cannabis, illegal in New York during the study period) or tested positive on urine drug screening for cannabis, amphetamines, barbiturates, benzodiazepines, cocaine, 3-4 methylenedioxymethamphetamine, opiates, or phencyclidine. Psychiatric and substance use exclusions were designed to minimize conditions that could confound cognitive performance. The TBI exclusions were implemented to avoid structural abnormalities that could interfere with image processing. The MRI contraindication exclusion was implemented for participant safety. Study procedures have been reported in detail.^38,39,41,42,43^ Collection of demographic information is detailed in eMethods 1 in Supplement 1.

Race and ethnicity data, although not a focus of this study, were collected to characterize the diversity and representativeness of the sample and were required by the funding agencies. Options self-identified by participants included American Indian or Alaska Native, Asian, Black or African American, Native Hawaiian or Pacific Islander, or White. Ethnicity was self-identified as Hispanic or non-Hispanic.

### RHI Exposure

RHI exposure was defined as the number of soccer headings over the prior 12 months, estimated using HeadCount, a structured survey validated in multiple independent cohorts against sideline observation and daily RHI reporting.^44,45,46^ The HeadCount survey uses a series of questions on soccer play and RHI designed and calibrated to account for variations in play year-round and to capture RHI from sources other than soccer heading.^44,45^ Due to the skew of RHI, we performed a sensitivity analysis in which RHI rank was the exposure variable. Ties in the rank list were handled by taking the mean ordered rank; for example, if the first 15 values were tied, these 15 values were assigned rank 8.

### Image Acquisition and Processing

3T MRI, including diffusion tensor imaging and/or neurite orientation dispersion and density imaging (DTI/NODDI), was performed and underwent standard preprocessing. Details are provided in eMethods 2 in Supplement 1.

### GWI Characterization

**Figure 1.**
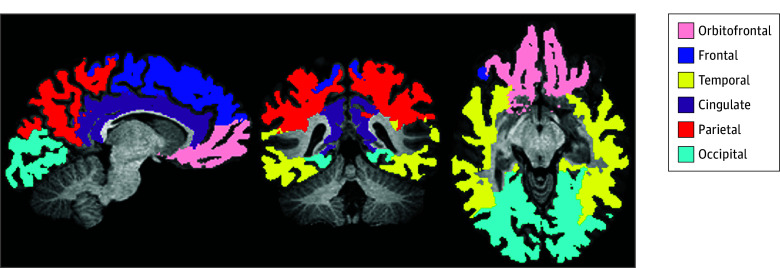
Lobar Segmentation Includes voxels 5 mm from Freesurfer 7–defined orbitofrontal gray matter–white matter interface (GWI) in white matter and 5 mm from GWI in gray matter. Reproduced from Song et al.^37^

Sharpness of DTI/NODDI metric transition at GWI was characterized by the slope of their profile (eFigure 1 in Supplement 1). We calculated FA, axial diffusivity (AD), orientation dispersion index (ODI), and intracellular volume fraction (ICVF) slope aggregated across 6 brain regions (cingulate, frontal, occipital, orbitofrontal, parietal, and temporal), as previously reported in healthy aging (Figure 1).^36,37^ The GWI slope calculation is detailed in eMethods 3 in Supplement 1.

### Cognitive Performance

We focused on the International Shopping List (ISL) test (immediate recall) of verbal learning and memory administered using CogState battery^47,48^ and associated with soccer RHI in this soccer cohort.^40^ A list of 12 words was read aloud to the participant, who was immediately asked to freely recall the words. The outcome measure was the total number of correct responses across 3 trials. Other CogState performance measures (ISL delayed recall task, One Back Test, Two Back Test, Card Identification Task, and Groton Maze Task) were administered but not associated with soccer RHI (eTable 1 in Supplement 1), as previously reported,^40,43^ and therefore were not tested for mediation.

### Statistical Analysis

#### GWI Slope and RHI Association

Calculations were performed using R, version 4.1.1 (R Project for Statistical Computing).^49^ We fit the following linear model to the slope of each diffusion metric (FA, AD, ODI, or ICVF): Y_i_ = β_0_ + β_1_H_i_ + β_2_C_i_ + e_1i_, where Y_i_ denotes the GWI slope for participant I, β_0_ denotes the intercept that would represent the mean GWI slope measure in the context of the covariates but no RHI exposure, H_i_ denotes 12-month RHI at the time of imaging, coefficient β_1_ denotes the independent association of RHI with GWI slope, and e_1i_ is a random error term. Biological sex (as a binary variable), age, and lifetime concussion (a categorical variable of 0, 1, or ≥2) were included as covariates in vector C_i_, with corresponding vector of coefficients β_2_. To account for multiple comparisons (6 regions for each of 4 diffusion metrics), we applied the Bonferroni method (*P* = .05/24), considering 2-sided *P* = .002 as our threshold for statistical significance. Post-hoc analyses of interaction effects and additional covariates are detailed in eMethods 4 in Supplement 1.

#### Mediation Analysis

To evaluate whether GWI slope mediates the association of RHI with ISL immediate recall score within this cohort,^40^ we applied the framework of Baron and Kenny.^50^ We fit the following 2 additional regression models: ISL_i_ = γ_0_ + γ_1_H_i_ + γ_2_C_i_ + e_2i_ and ISL_i_ = δ_0_ + δ_1_Hi + δ_2_Y_i_ + δ_3_C_i_ + e_3i_, where ISL_i_ denotes the cognitive outcome for participant I; H_i_ and Y_i_ are as defined in the previous paragraph; C_i_ represents a vector of covariates as defined in the previous paragraph and includes biological sex (as a binary variable), age (as a continuous variable), and lifetime concussion (a categorical variable–0, 1, 2+); γ_0_ and δ_0_ are the intercepts in each model, respectively; γ_1_ quantifies the association of RHI (H_i_) with ISL after adjusting for C_i_ (ie, total effect of RHI on ISL), while δ_1_ denotes the same association but further controlling for the effect of the mediator Y_i_ (ie, direct effect of RHI); δ_2_ quantifies the association between Y_i_ and ISL_i_ after accounting for H_i_ and C_i_; γ_2_ and δ_3_ correspond to the independent effects of the covariates in each model, respectively; and e_2i_ and e_3i_ are random error terms.

Mediation by GWI slope (Y_i_) was calculated from these 2 equations, where the indirect effect of the association of RHI with cognitive outcomes, mediated through GWI slope, was computed as β_1_ × δ_2_, and significance was determined using the Sobel test.^51^ Since the mediation analysis focused on just 1 mediator (orbitofrontal GWI FA slope), no adjustment was made for multiple testing, and 2-sided *P* = .05 was our threshold for statistical significance.

## Results

We included 352 amateur soccer players (median age, 23.0 years [range, 18.0-53.0 years]; mean [SD] age, 25.6 [7.5] years; 109 [31.0%] female and 243 [69.0%] male) from the New York City area. Of the 314 soccer players who identified their race, 1 (3.2%) was American Indian or Alaska Native; 62 (19.7%), Black or African American; 5 (1.6%), Native Hawaiian or Other Pacific Islander; and 228 (72.6%), White. Of the 336 who identified their ethnicity, 82 (24.4%) were Hispanic. Estimates of RHI exposure ranged from 0 to 22 838 in the past 12 months, with a median of 677.5. Age, biological sex, race, ethnicity, years of education, and concussion history did not differ significantly by RHI exposure level (Table 1).

**Table 1.** Study Sample Demographic Characteristics by RHI Exposure Quartile^a^

Characteristic	Participants^b^			
	1st Quartile (n = 88)	2nd Quartile (n = 88)	3rd Quartile (n = 88)	4th Quartile (n = 88)
Age, median (range), y	27 (18-53)	24 (18-50)	22 (18-52)	21 (18-38)
Biological sex				
Male	47/88 (53.4)	59/88 (67.0)	70/88 (79.5)	67/88 (76.1)
Female	41/88 (46.6)	29/88 (33.0)	18/88 (20.5)	21/88 (23.9)
Educational attainment, median (range), y	16 (7-20)	16 (12-20)	15 (10-20)	15 (12-19)
Race				
American Indian or Alaska Native	0/79	0/80	1/82 (1.2)	0/73
Asian	3/79 (3.8)	4/80 (5.0)	7/82 (8.5)	4/73 (5.5)
Black or African American	7/79 (8.9)	16/80 (20.0)	16/82 (19.5)	23/73 (31.5)
Native Hawaiian or Pacific Islander	1/79 (1.3)	1/80 (1.3)	1/82 (1.2)	2/73 (4.1)
White	68/79 (86.1)	59/80 (73.8)	57/82 (69.5)	44/73 (60.3)
Declined to answer	9	8	6	15
Ethnicity				
Hispanic	12/85 (14.1)	19/85 (21.2)	24/84 (28.6)	27/81 (33.3)
Non-Hispanic	73/85 (85.9)	66/85 (77.6)	60/84 (71.4)	54/81 (66.7)
Declined to answer	3	3	4	7
12-mo RHI count, median (range)	104 (0-287)	486 (296-677)	1147 (678-1863)	3133 (1877-22 838)
Concussions, No.				
0	57	54	56	55
1	17	17	15	10
≥2	14	17	17	23
ISL immediate recall task, No. correct across 3 trials, mean (SD)^c^	27.1 (3.8)	26.5 (3.7)	25.5 (3.8)	25.4 (3.9)

Abbreviations: ISL, International Shopping List; RHI, repetitive head injury.

^a^ Quartile 1 was rank 8.0 to 88.0; quartile 2, 89.5 to 176.0; quartile 3, 177.0 to 264.0; and quartile 4, 265.5 to 352.0.

^b^ Data are presented as number/total number (percentage) of participants unless otherwise indicated.

^c^ The ISL test is described in the Cognitive Performance subsection of the Methods section.

### GWI Slope Associations With RHI

**Figure 2.**
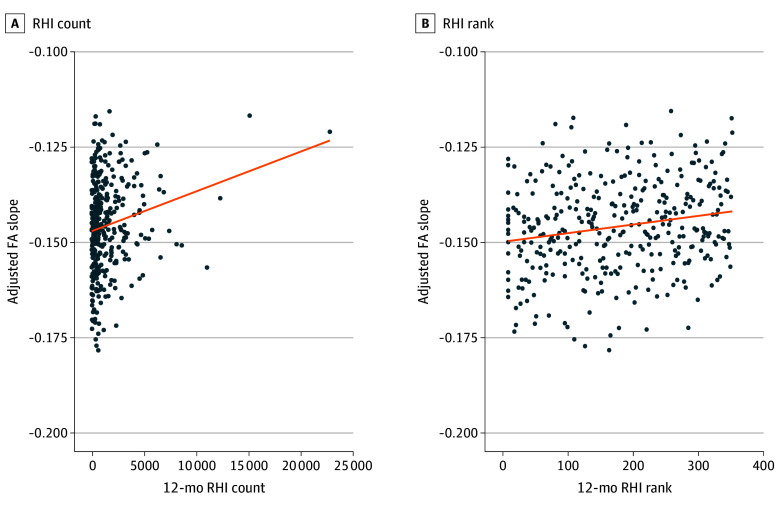
Orbitofrontal Adjusted Fractional Anisotropy (FA) Slope vs Repetitive Head Impacts (RHI) A, The association between orbitofrontal region FA slope and RHI count (β_1_ = 0.000001; *P* < .001). B, The association between orbitofrontal region FA slope and RHI rank (β_1_ = 0.000023; *P* = .001).

Using the regression model, greater RHI was associated with less sharp FA slope at the GWI in the orbitofrontal region (estimate, 0.000001; *P* < .001) while accounting for age, biological sex, and prior concussion (Table 2 and Figure 2). No associations were observed for other regions (Table 2 and eFigure 2 in Supplement 1). FA slope was associated with age, as previously reported,^37^ and was not associated with biological sex or concussion history (eTable 2 in Supplement 1). There were no significant interactions of biological sex and RHI or age and RHI (eTable 3 in Supplement 1). In sensitivity analyses to account for extreme RHI values, RHI rank was associated with less sharp orbitofrontal GWI FA slope (estimate, 0.000023; *P* = .001) (Table 2, Figure 2, and eFigure 3 in Supplement 1). RHI exposure was not associated with ODI slope, AD slope, or ICVF slope for any of the 6 regions (eTables 4-6 in Supplement 1). Additional covariates (ie, educational level, self-reported medical history, alcohol use, and depression and anxiety symptoms) were not statistically significant (eTables 7 and 8 in Supplement 1).

**Table 2.** Linear Model Results for Fractional Anisotropy Slope^a^

Region	12-mo RHI count		12-mo RHI rank	
	β_1_	*P* value^b^	β_1_	*P* value^b^
Cingulate	−0.0000002	.49	−0.000001	.87
Frontal	0.0000006	.03	0.000011	.08
Occipital	0.0000004	.30	0.000013	.10
Orbitofrontal	0.000001	<.001	0.000023	.001
Parietal	0.00000001	.63	0.000006	.38
Temporal	0.0000005	.02	0.000010	.05

Abbreviation: RHI, repetitive head injury.

^a^ Linear models were fit to both 12-month RHI count and 12-month RHI rank. β_1_ Refers to the estimate of RHI association with fractional anisotropy slope.

^b^ *P* < .002 was considered statistically significant.

### Orbitofrontal GWI FA Slope and Mediation of the Association of RHI With Cognitive Performance

**Figure 3.**
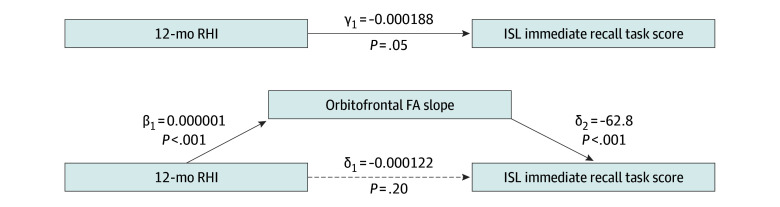
Orbitofrontal Fractional Anisotropy (FA) Slope Mediation Analysis γ_1_ Is the estimate of direct total effect of the association of repetitive head impact (RHI) with International Shopping List (ISL) (outcome measure); δ_1_ is the indirect effect of the association of RHI with ISL; δ_2_ is the estimate of orbitofrontal FA slope effect on ISL; β_1_ is the estimate of RHI effect on orbitofrontal FA slope (mediator); and β_1_ × δ_2_ is the indirect effect of orbitofrontal FA slope (mediator) on ISL (outcome measure). All regression coefficients reported are from equations shown in the Statistical Analysis subsection of the Methods section.

Using regression models, total effect for the association of RHI exposure with ISL immediate recall task scores was significant (estimate, γ_1_ = 0.000188; *P* = .05) (Figure 3). The indirect effect for the association of RHI with ISL performance, mediated through orbitofrontal FA slope, was significant (estimate, γ_1_ × δ_2_ = −0.000064; *P* = .006), while the direct effect for the association of RHI with ISL immediate recall performance was not significant (estimate, δ_1_ = −0.000122; *P* = .20) (Figure 3). Thus, orbitofrontal FA slope mediated the association of greater RHI with poorer immediate recall ISL task score (Figure 3), consistent with a mechanistic role of GWI slope in the adverse RHI-ISL association. Age, biological sex, and concussion history were accounted for in the models.

## Discussion

In this cross-sectional study, high RHI exposure was associated with blunting of the naturally sharp microstructural transition at the orbitofrontal GWI but not in other regions. This pattern indicates specific susceptibility of the orbitofrontal GWI to RHI effects, consistent with finite element models that localize the greatest tissue strain during soccer heading to the orbitofrontal region.^34^ Also notable is that the floor of the anterior cranial fossa, on which the orbitofrontal region rests, is located in line with the approximate soccer ball trajectory during heading.^34^ The orbitofrontal findings may therefore reflect a contrecoup injury by which brain acceleration leads to impact of orbitofrontal cortex on the irregular surface of the floor of the anterior cranial fossa.

Concentration of RHI effects at the GWI was consistent with shear force effects that arise due to tissue density differences and consequent differences in acceleration of white and gray matter at the GWI.^19^ Susceptibility of the GWI to shear force trauma is supported by numerous lines of evidence, including the location of microhemorrhage in TBI,^28,33^ postmortem histology localizing astrogliosis to the GWI after RHI in sheep^24^ and pigs,^21,22,23^ and postmortem studies of RHI-exposed athletes and veterans.^25,52^ Furthermore, TBI-induced astrogliosis in rats is linked to changes in cortical gray matter FA,^53^ linking astrogliosis near the GWI with FA. In the present study, attenuation of FA slope sharpness in association with RHI exposure could have occurred due to astrogliosis and white matter myelin perturbations at the GWI.

We failed to reject our null hypotheses for the association of RHI with the GWI slope of other diffusion metrics (AD, ODI, and ICVF). FA slope may detect the microstructural features of RHI-associated injury, which other metrics do not. Alternatively, if effects of RHI on other diffusion metrics are of lower magnitude, it is possible that we did not have sufficient power to detect these associations. Power for assessment of NODDI metrics in this study was limited by a somewhat small sample size (338 vs 352 usable datasets), although this difference in sample may not fully explain the results. We note that prior studies of RHI have overwhelmingly reported an association of RHI with FA, with few studies reporting on other diffusion metrics.^14^ This difference in detection by diffusion MRI metrics underscores that while all derive from the same MRI signal, different metrics capture different biophysical features of diffusion that, in turn, reflect different microstructural characteristics of tissue. ODI, for example, estimates the angular variability of neurites in each voxel, which may not be sensitive to loss of myelin and axons known to occur in injury contexts.

Prior dMRI studies of RHI have generally used region or tract of interest or voxel-wise analyses to extract average measures of diffusion metrics from lobar and deep white matter,^14,40,54^ typically excluding gray matter and juxtacortical white matter voxels, including the GWI, from analysis due to concerns for partial volume effects, misregistration, or low signal-to-noise ratio. To date, RHI-associated changes to GWI microstructure may have gone undetected or underestimated with in vivo imaging. The present study used an approach that is not limited by and accounts for potential partial volume effects (see below) on individual voxels near the GWI. Because the GWI slope measure is determined in each individual, it does not depend on image registration across individuals or to a template. This approach enables the investigation of RHI-related brain tissue changes that have previously been inaccessible in vivo.

We found that lower orbitofrontal GWI sharpness (lower slope of FA) was associated with poorer performance on the ISL immediate recall task. For the ISL immediate recall task, the participant listens to a list of words read aloud and then recites as many as they can recall to the examiner. Importantly, the ISL uses a list of words linked by context (a grocery shopping list). The ISL is typically conceptualized as the first step in a verbal memory task, which is completed with delayed recall after 20 minutes.^47,55^ The orbitofrontal region, however, is not considered a key substrate of verbal learning and memory functions. Because the orbitofrontal region is implicated in executive function, planning, strategy, and attention, it is plausible that effective planning and verbal learning strategies depend on orbitofrontal functions.^56^ It is also plausible that orbitofrontal GWI injury leads to poorer learning strategies, in turn reflected in lower ISL score.

Identification of brain injury mechanisms and in vivo detection in humans is a priority of current research. Prior studies^38,39,40^ have reported associations of soccer RHI with dMRI and with cognitive performance. Although RHI is independently associated with both brain structure (imaging) and brain function, these 2 distinct associations do not establish that the structural changes mediate the association between RHI and objective cognitive performance. Identifying such a causal link would point more specifically to the mechanism driving adverse cognitive effects of RHI. We performed mediation analysis to assess whether GWI microstructure effects mediated the association between RHI and cognitive performance. Our result supports the mechanistic role of orbitofrontal GWI FA slope.

Future studies should investigate this in vivo mechanistic indicator. Clinical research could use dMRI GWI findings to identify RHI exposure levels linked to functional effects and to inform protective interventions. In this study, the cognitive effects mediated by dMRI were significant but did not reach a magnitude that reflected clinical impairment. Future longitudinal studies could probe domains, such as occupational, social, or academic functioning, to assess the impact on current or future functioning in these contexts. Translational studies could explore mechanisms underlying GWI diffusion effects to develop therapies and use the GWI slope as a proxy end point in preclinical and human studies. While further investigation is needed, these findings suggest that GWI slope may serve as a promising in vivo marker of RHI-related pathology, offering potential clinical utility in the detection of pathologic change that could ultimately be associated with current dysfunction or future disorders such as CTE. Clinical implementation to identify specific patient groups will require future studies, potentially including comparison with postmortem findings and or animal models, and identification of specific thresholds that would indicate presence of pathology or clinical features of disease.

### Limitations

This study has some limitations. The possibility of bias due to partial volume effects cannot be entirely excluded. However, our approach mitigated the impact of potential voxel-level partial volume effects near the GWI by not relying on intensity at any given voxel. Instead, we computed change of the diffusion parameter across multiple voxels along linear trajectories spanning the GWI. The polynomial fit used to derive the slope incorporated the variance of the diffusion metric at each distance from the GWI, ensuring accurate propagation of residual noise. While error levels are low,^36^ this fitting method helps maintain robustness in the presence of minor noise. Thus, the dMRI signal slope variations that we observed primarily reflect diffusion differences across the GWI, without bias due to partial volume effects from individual voxels near the GWI.

If GWI slope depended on the precise anatomic GWI location based on segmentation and parcellation, misregistration could be a significant concern. Our method, however, did not solely rely on an anatomic localization of the GWI. The GWI defined by FreeSurfer brain imaging software, version 7.0.6 (Laboratory for Computational Neuroimaging at the Athinoula A. Martinos Center for Biomedical Imaging), was used to bin voxels to generate the GWI profile (eFigure 1 in Supplement), which was self-organizing. The GWI slope calculation was mainly driven by the long linear segment of the GWI profile in the vicinity of the FreeSurfer-defined GWI, making it insensitive to shifts from misregistration. In this way, our method was self-organizing and more robust than other GWI region of interest studies that have placed the region of interest adjacent to the GWI and relied heavily on a presumption of exact registration of the GWI in each individual brain.^31,32^

Our exposure measurement was based on self-report and has been validated in multiple studies to reliably rank players with respect to relative heading exposure.^44,45^ The survey was not designed to quantify absolute number or characterize the severity of individual impacts. The estimates of ranked headings across players were suited to our context, which addresses a biological hypothesis on the way greater amounts of heading will manifest in neuroimaging and cognitive performance. We acknowledge that future studies aimed at validating exposure thresholds, particularly for use in risk mitigation strategies, should further assess whether the exposure measure can reliably and reproducibly capture absolute exposure levels.

The RHI count data were right-skewed, in part due to some participants engaging in frequent soccer heading drills, which involve numerous high-repetition sets across multiple practice days per week. Moreover, our sensitivity analysis showed a linear association with RHI rank that reduced the need for precise RHI counts. While RHI estimates prior to the 12-month period during which we estimated RHI are not available for this cohort, our team showed in a previous study that age of first exposure to soccer RHI did not moderate the adverse association of RHI with outcomes.^57^ Future studies can address the role of cumulative lifetime exposure.

Participants were not active in other collision sports during the study. Although prior contact sport exposure cannot be excluded, it would be unlikely to systematically covary with the study measures.

We did not include formal performance validity testing. While it is possible that suboptimal engagement contributed to performance, the distribution of performance in the sample in our study suggests that this was not the case.

While key neurological conditions, such as dementia and moderate-to-severe TBI, acute (within 3 months) or persistent symptomatic concussion (mild TBI) were excluded, residual confounding by unmeasured factors, including family history of neurological disease, which was not assessed, remains possible. We performed post-hoc analysis assessing potential confounders (ie, educational level, self-reported medical history, alcohol use, and depression and anxiety symptoms), and none were found to be significant. Future studies incorporating genetic or familial risk factors may further clarify individual susceptibility. DTI, while sensitive, lacks specificity and must be considered in context, as it was applied in the present study.

Last, 31.0% of the total cohort was female, which may limit the generalizability of findings. However, the cohort reflects the predominantly male amateur player population in New York and, in fact, oversampled female players.

## Conclusions

In this cross-sectional study of adult amateur soccer players, we found that RHI due to soccer heading was associated, in an exposure-dependent fashion, with attenuation of the normally sharp microstructural transition at the cortical GWI of the orbitofrontal region. This imaging feature in turn mediated the previously reported adverse association^38,40^ of greater RHI with worse performance on the ISL immediate recall task. Future studies of soccer RHI should use cognitive tests optimized to test orbitofrontal function, such as executive function, and explore the GWI measure as a tool to characterize mechanisms of adverse RHI effects in vivo.

## Notes

Supplement 1. **eMethods 1.** Race and Ethnicity **eMethods 2.** Scanning Procedure **eMethods 3.** Computation of GWI Slope **eMethods 4.** Post-Hoc Analysis **eFigure 1.** Schematic for Slope of FA Calculation in 1 Region for 1 Participant **eFigure 2.** Adjusted FA Slope vs 12-mo RHI Count **eFigure 3.** Sensitivity Analysis: Adjusted FA Slope vs 12-mo RHI Rank **eTable 1.** CogState Battery Linear Associations With 12-mo RHI **eTable 2.** Detailed Linear Model Results for FA Slope With Covariate Effect Estimates **eTable 3.** Linear Model Results for Orbitofrontal FA Slope With Interaction Effects **eTable 4.** Linear Model Results for ICVF Slope **eTable 5.** Linear Model Results for ODI Slope **eTable 6.** Linear Model Results for AD Slope **eTable 7.** Additional Study Demographic Characteristics **eTable 8.** Post-Hoc Analysis: Linear Model Results for Orbitofrontal FA Slope Association RHI With Additional Covariate Effect Estimates

Supplement 2.
Data Sharing Statement
