# Supplementary material for: Orbitofrontal Gray-White Interface Injury and the Association of Soccer Heading With Verbal Learning
Source: JAMA Netw Open. 2025 Sep 18;8(9):e2532461. doi: 10.1001/jamanetworkopen.2025.32461 (PMC12447236; doi:10.1001/jamanetworkopen.2025.32461)
Supplement: Supplement 1. — eMethods 1. Race and Ethnicity eMethods 2. Scanning Procedure eMethods 3. Computation of GWI Slope eMethods 4. Post-Hoc Analysis eFigure 1. Schematic for Slope of FA Calculation in 1 Region for 1 Participant eFigure 2. Adjusted FA Slope vs 12-mo RHI Count eFigure 3. Sensitivity Analysis: Adjusted FA Slope vs 12-mo RHI Rank eTable 1. CogState Battery Linear Associations With 12-mo RHI eTable 2. Detailed Linear Model Results for FA Slope With Covariate Effect Estimates eTable 3. Linear Model Results for Orbitofrontal FA Slope With Interaction Effects eTable 4. Linear Model Results for ICVF Slope eTable 5. Linear Model Results for ODI Slope eTable 6. Linear Model Results for AD Slope eTable 7. Additional Study Demographic Characteristics eTable 8. Post-Hoc Analysis: Linear Model Results for Orbitofrontal FA Slope Association RHI With Additional Covariate Effect Estimates [file jamanetwopen-e2532461-s001.pdf]

Supplementary Online Content

Song JY, Fleysher R, Ye K, et al. Orbitofrontal gray-white interface injury from soccer heading and verbal learning outcomes. *JAMA Netw Open.* 2025;8(9):e2532461. doi:10.1001/jamanetworkopen.2025.32461

eMethods 1. Race and Ethnicity
eMethods 2. Scanning Procedure
eMethods 3. Computation of GWI Slope
eMethods 4. Post-Hoc Analysis
eFigure 1. Schematic for Slope of FA Calculation in 1 Region for 1 Participant
eFigure 2. Adjusted FA Slope vs 12-mo RHI Count
eFigure 3. Sensitivity Analysis: Adjusted FA Slope vs 12-mo RHI Rank
eTable 1. CogState Battery Linear Associations With 12-mo RHI
eTable 2. Detailed Linear Model Results for FA Slope With Covariate Effect Estimates
eTable 3. Linear Model Results for Orbitofrontal FA Slope With Interaction Effects
eTable 4. Linear Model Results for ICVF Slope
eTable 5. Linear Model Results for ODI Slope
eTable 6. Linear Model Results for AD Slope
eTable 7. Additional Study Demographic Characteristics
eTable 8. Post-Hoc Analysis: Linear Model Results for Orbitofrontal FA Slope Association RHI With Additional Covariate Effect Estimates

This supplementary material has been provided by the authors to give readers additional information about their work.

© 2025 Song JY et al. *JAMA Network Open*.

eMethods 1. Race and Ethnicity

Demographic information related to race and ethnicity was self-reported by participants using an electronic form. Race and ethnicity were not a focus of this study. However, collection of these data allowed us to characterize the diversity and representativeness of the sample and was required by the funding agencies. Race options were (alphabetical order): "American Indian or Alaska Native," "Asian," "Black or African American," "Native Hawaiian or Pacific Islander," "White". Additional options included: "More than one race", "Other" and "Decline to report". Ethnicity options were "Hispanic," "Non-Hispanic," or "Decline to report.

eMethods 2. Scanning Procedure

All data were collected on the same 3.0T Philips Achieva TX scanner using a 32-channel head coil (Philips Medical Systems, Best, The Netherlands). **An auxiliary field map** was acquired using FOV=250mm, 3.0 mm^3 isotropic resolution, TR=26ms, TE/ΔTE= 2.5/2.3ms, α = 26° and SENSE factor = 2 (anterior-posterior) x 2 (head-foot). The field map is used to correct small susceptibility-induced distortions in T1-weighted and echo-planar scans. **3D T1-weighted (T1W)** magnetization-prepared rapid acquisition of gradient echo imaging was performed with TR/TE/TI = 9.9/4.6/900 ms, α =8°, SENSE factor along SI/RL= 2/2.6, 1 mm^3 isotropic resolution, 240 × 188 × 220 matrix. **Neurite Orientation Dispersion Density Imaging (NODDI)** was acquired using 2D single-shot spin-echo echo-planar-imaging (EPI) with 10 interleaved volumes at b=0 s/mm^2 and three diffusion-weighted shells [6 directions at b=300 s/mm^2, 32 at b=800 s/mm^2 and 60 at b=2000 s/mm^2], TE = 95ms, TR = 10s, 2 mm^3 isotropic resolution, 128 × 120 matrix, 70 slices, SENSE factor=2.8. **Diffusion Tensor Imaging (DTI)** is a subset with b=0 s/mm^2 and b=800 s/mm^2. **Preprocessing:** T1W, DTI and NODDI undergo preprocessing, including skull stripping, correction for motion, eddy currents and EPI distortion using FreeSurfer and FSL tools [48]. DTI metrics are obtained by fitting the b=0 and b=800 images [FDT-DTIFIT [49, 50]]. NODDI metrics are derived using fit [AMICO (Accelerated Microstructure Imaging via Convex Optimization [51]] to characterize intra- and extra-cellular diffusion. **Spatial Registration:** We use rigid body transformation to register DTI and NODDI metric maps to each subject's T1W image as reported previously [52] but enhanced by Advanced Neuroimaging Tools (ANTs) [53] and using the Mean Absolute Regional LINear correlation Algorithm (MARLINA) cost function [54]. **Segmentation:** The GWI and 6 brain regions (cingulate, frontal, occipital, orbitofrontal, parietal, and temporal) were defined by FreeSurfer 7 segmentation [55] (Figure 1). **Inspection:** All images were visually inspected to ensure they were free of motion or hardware artifacts, with successful skull stripping and registration. Any inaccuracies in skull stripping were manually corrected, and the data was reprocessed.

eMethods 3. Computation of GWI Slope

To compute the GWI slope [36, 37], we first compute the shortest Euclidean distance to the FreeSurfer 7 [55] defined GWI for each voxel (Supplemental Figure 1. (ii)). The orthogonal vector, derived from this shortest distance, defines the direction along which slope is calculated. Positive distances from a voxel to the FreeSurfer-defined GWI denote the GM "side" of the GWI, while negative distances indicate the WM "side." All voxels are sorted according to their shortest distance from the GWI (Supplemental Figure 1. (iii)). For each distance bin, we find the average DTI/NODDI value and its standard deviation (Supplemental Figure 1. (iv)). The aggregate slope of a DTI/NODDI metric across each brain region is derived from a 7th degree polynomial fit (gnuplot [56]) to the *average* measure at each distance bin from the GWI (Supplemental Figure 1. (v)). The aggregate slope is defined at the nearest point of local maximum slope magnitude that is same sign as the slope at the FreeSurfer-defined GWI (x=0) (Supplemental Figure 1. (v)). Note that the point of maximal slope is not necessarily at the FreeSurfer-defined GWI. The sign of the GWI slope is due to our definition of distance from the GWI, where GM is represented as the positive distance, and WM as the negative distance. The steepness of the slope, which is our measure of interest, represents the sharpness of the diffusion parameter transition, regardless of sign.

© 2025 Song JY et al. *JAMA Network Open*.

eMethods 4. Post-Hoc Analysis

In a post-hoc analysis to explore interaction effects, we re-evaluated the association of RHI with GWI slope by including an interaction effect between age with RHI count and biological sex with RHI in (1). We also conducted analyses that adjusted for additional covariates including educations (years), self-reported medical history (high blood pressure, heart disease, diabetes, stroke), self-reported alcohol use (average alcoholic drinks per week, maximum alcoholic drinks on one occasion), and depression and anxiety symptoms quantified using the Patient-Reported Outcomes Measurement Information System (PROMIS), as previously detailed [43, 52, 53].

eFigure 1. Schematic for Slope of FA Calculation in 1 Region for 1 Participant

Same procedure would be applied to all GWI slope measures. i) FA map shows natural sharp change in FA at the GWI (e.g., red box). ii) Magnification of the red box delineated in (i). For each voxel (e.g., pink square), the shortest Euclidean distance to the GWI (green dotted line) is calculated; the orthogonal vector, defined by shortest distance, will be the orientation along which we will compute the slope of FA. Positive distance is on the GM "side" of the GWI (dark), and negative distance is on the WM "side" (lighter shade of gray). iii) All voxels are binned by distance to the GWI; this can be done for each region (e.g., orbitofrontal, shaded pink in panel iii (a)). Within each bin, the average FA (red circle) and standard deviation are calculated. iv) Shows real data from one participant. This plot presents average and standard deviation of FA for each bin (y-axis) vs. the distance from the FreeSurfer-defined GWI (x-axis) corresponding to the bin. Note the small error bars, indicating minimal noise. v) We fit a 7th order smoothing polynomial (black curve) to the points in (iv), taking errors into account. The GWI slope of FA (green arrow) is taken as the maximum slope of the fitted polynomial near x=0 (i.e., the FreeSurfer-defined GWI). Figure from previous methods paper [37].

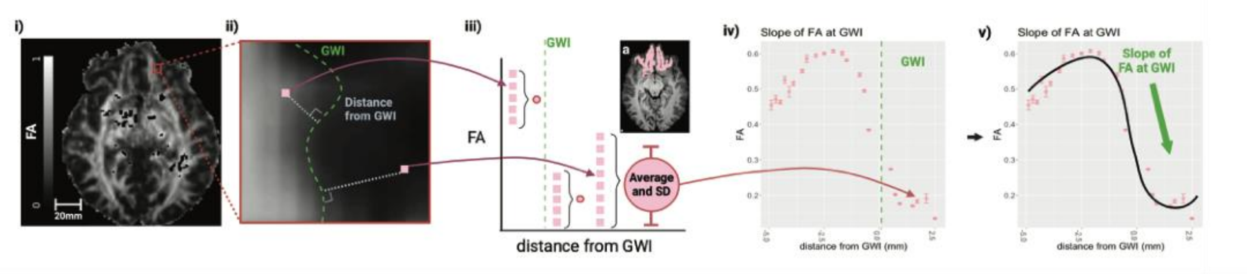

eFigure 2. Adjusted FA Slope vs 12-mo RHI Count

A) The association between cingulate region FA slope and RHI count (β_1 = -0.0000002, p = 0.49) **B)** The association between frontal region FA slope and RHI count(β_1 = 0.0000006, p = 0.03) **C)** The association between occipital region FA slope and RHI count (β_1 = 0.000000362, p = 0.30) **D)** The association between parietal region FA slope and RHI count (β_1 = 0.000000142, p = 0.63) **E)** The association between temporal region FA slope and RHI count (β_1 = 0.000000536, p = 0.02)

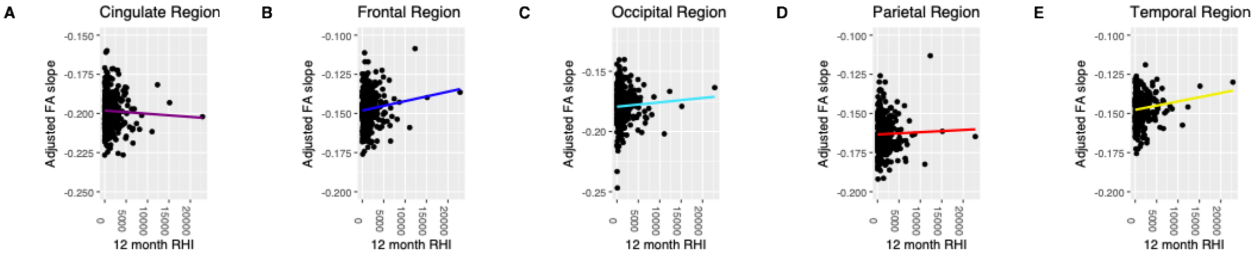

© 2025 Song JY et al. *JAMA Network Open*.

eFigure 3. Sensitivity Analysis: Adjusted FA Slope vs 12-mo RHI Rank

A) The association between cingulate region FA slope and RHI rank (β_1 = -0.0000011, p = 0.87) **B)** The association between frontal region FA slope and RHI rank (β_1 = 0.0000111, p = 0.08) **C)** The association between occipital region FA slope and RHI rank (β_1 = 0.000013, p = 0.10) **D)** The association between parietal region FA slope and RHI rank (β_1 = 0.00000595, p = 0.38) **E)** The association between temporal region FA slope and RHI rank (β_1 = 0.0000105, p = 0.05)

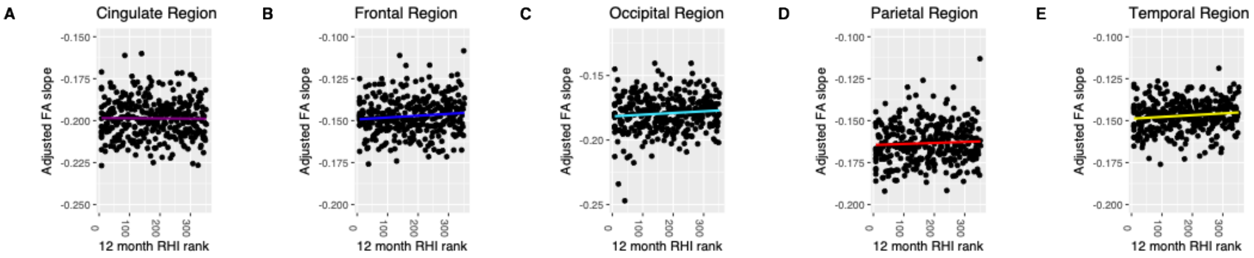

© 2025 Song JY et al. *JAMA Network Open*.

eTable 1. CogState Battery Linear Associations With 12-mo RHI

To ensure a comprehensive evaluation, these supplemental analyses were conducted to confirm the absence of associations previously found to be null, expect with the ISL immediate recall task. Concussion history, biological sex and age were included as covariates.

Cognitive Test	Term	Estimate	P-value
**Card Identification (reaction time)**	(Intercept)	2.70161666	<0.0001
**Card Identification (reaction time)**	Age	-0.0015147	0.6211877
**Card Identification (reaction time)**	Biological Sex (Male)	-0.0101248	0.8367072
**Card Identification (reaction time)**	RHI count	0.00000435	0.67665288
**Card Identification (reaction time)**	Concussion (1)	-0.1104185	0.07209439
**Card Identification (reaction time)**	Concussion (2+)	0.00077755	0.98929489
**Groton Maze Chase Task (# of errors)**	(Intercept)	1.99843029	<0.0001
**Groton Maze Chase Task (# of errors)**	Age	-0.0187796	<0.0001
**Groton Maze Chase Task (# of errors)**	Biological Sex (Male)	-0.0272162	0.60792943
**Groton Maze Chase Task (# of errors)**	RHI count	-0.0000153	0.17406394
**Groton Maze Chase Task (# of errors)**	Concussion (1)	0.05617851	0.39587672
**Groton Maze Chase Task (# of errors)**	Concussion (2+)	0.02890764	0.64414364
**ISL Immediate Recall (# correct)**	(Intercept)	27.6460243	<0.0001
**ISL Immediate Recall(# correct)**	Age	-0.0037407	0.89354691
**ISL Immediate Recall(# correct)**	Biological Sex (Male)	-1.8032531	<0.0001
**ISL Immediate Recall(# correct)**	RHI count	-0.0001877	0.04933681
**ISL Immediate Recall(# correct)**	Concussion (1)	-0.4087163	0.46461322
**ISL Immediate Recall(# correct)**	Concussion (2+)	0.74981779	0.15664831
**ISL Delayed Recall(# correct)**	(Intercept)	10.2435986	<0.0001
**ISL Delayed Recall(# correct)**	Age	0.00731674	0.56244617

© 2025 Song JY et al. *JAMA Network Open*.

**ISL Delayed Recall(# correct)**	Biological Sex (Male)	-1.3251289	<0.0001
**ISL Delayed Recall(# correct)**	RHI count	-0.0000444	0.30251392
**ISL Delayed Recall(# correct)**	Concussion (1)	-0.1270036	0.61492245
**ISL Delayed Recall(# correct)**	Concussion (2+)	-0.0329944	0.89011867
**One Back Test (accuracy)**	(Intercept)	1.30457098	<0.0001
**One Back Test (accuracy)**	Age	-0.0016629	0.41635898
**One Back Test (accuracy)**	Biological Sex (Male)	-0.0352565	0.28364465
**One Back Test (accuracy)**	RHI count	-0.00000921	0.16823288
**One Back Test (accuracy)**	Concussion (1)	-0.0583339	0.16593016
**One Back Test (accuracy)**	Concussion (2+)	0.08648163	0.02515631
**Two Back Test (accuracy)**	(Intercept)	1.24017544	<0.0001
**Two Back Test (accuracy)**	Age	-0.0024106	0.23833968
**Two Back Test (accuracy)**	Biological Sex (Male)	-0.0091024	0.78098401
**Two Back Test (accuracy)**	RHI count	-0.00000564	0.41808208
**Two Back Test (accuracy)**	Concussion (1)	-0.0718484	0.07904393
**Two Back Test (accuracy)**	Concussion (2+)	0.04243682	0.27225953

© 2025 Song JY et al. *JAMA Network Open*.

eTable 2. Detailed Linear Model Results for FA Slope With Covariate Effect Estimates

Linear models were fit to 12-month RHI count as described in Equation 1.

Region of GWI FA slope measure calculation	Term	Effect estimate	P-value
cingulate	(Intercept)	-0.19817	7E-223
cingulate	Age	0.000501	8.32E-09
cingulate	Biological Sex (Male)	0.001062	0.4353
cingulate	RHI count	-2E-07	0.485689
cingulate	Concussion (1)	-0.0001	0.95258
cingulate	Concussion (2+)	0.001402	0.382547
frontal	(Intercept)	-0.14814	3E-189
frontal	Age	0.000426	2.25E-07
frontal	Biological Sex (Male)	0.003141	0.015515
frontal	RHI count	6E-07	0.029432
frontal	Concussion (1)	-0.00104	0.517616
frontal	Concussion (2+)	0.001237	0.417503
occipital	(Intercept)	-0.17941	6.6E-182
occipital	Age	0.000496	2.15E-06
occipital	Biological Sex (Male)	0.005608	0.000757
occipital	RHI count	3.62E-07	0.302737
occipital	Concussion (1)	-0.00088	0.668982
occipital	Concussion (2+)	0.002373	0.223786
orbital	(Intercept)	-0.14705	2.2E-174
orbital	Age	0.000498	4.88E-08
orbital	Biological Sex (Male)	0.002536	0.077001
orbital	RHI count	1.04E-06	0.000679
orbital	Concussion (1)	-0.00088	0.622569
orbital	Concussion (2+)	-0.0009	0.595311
parietal	(Intercept)	-0.16339	1.6E-193
parietal	Age	0.000476	6.72E-08
parietal	Biological Sex (Male)	0.001674	0.226436
parietal	RHI count	1.42E-07	0.627673
parietal	Concussion (1)	0.000503	0.77054
parietal	Concussion (2+)	0.001313	0.420874
temporal	(Intercept)	-0.14767	1.5E-214
temporal	Age	0.000324	2.05E-06
temporal	Biological Sex (Male)	0.00179	0.09666
temporal	RHI count	5.36E-07	0.019489
temporal	Concussion (1)	-0.00264	0.049721
temporal	Concussion (2+)	-0.0013	0.306674

© 2025 Song JY et al. *JAMA Network Open*.

© 2025 Song JY et al. *JAMA Network Open*.

eTable 3. Linear Model Results for Orbitofrontal FA Slope With Interaction Effects

Linear models were fit to 12-month RHI count as described in Equation 1 with additional age x RHI and sex x RHI interaction effects.

Term	Effect estimate	P-value
(Intercept)	-0.1473175	<0.0001
Age	0.00047117	<0.0001
Biological Sex (Male)	0.00000062	0.77531158
RHI count	0.00373625	0.0292834
Concussion (1)	-0.0008332	0.64388158
Concussion (2+)	-0.0009924	0.55959509
Age: RHI count	0.00000005	0.60855577
RHI count : Biological Sex (Male)	-0.00000084	0.21250445

© 2025 Song JY et al. *JAMA Network Open*.

eTable 4. Linear Model Results for ICVF Slope

Linear models were fit to both 12-month RHI count and 12-month RHI rank. β_1 refers to the estimate of RHI association with ICVF slope in Equation 1.

Regions	12 month RHI *count* estimate β_1	12 month RHI *count* estimate p-value	12 Month RHI *rank* estimate β_1	12 Month RHI *rank* estimate p-value
**cingulate**	7.55E-07	0.07	1.77E-05	0.07
**frontal**	7.29E-07	0.02	1.46E-05	0.06
**occipital**	5.5E-07	0.04	8.82E-06	0.17
**orbitofrontal**	8.34E-07	0.02	1.33E-05	0.11
**parietal**	8.48E-07	0.01	1.86E-05	0.02
**temporal**	9.99E-07	0.006	2.3E-05	0.008

© 2025 Song JY et al. *JAMA Network Open*.

eTable 5. Linear Model Results for ODI Slope

Linear models were fit to both 12-month RHI count and 12-month RHI rank. Bolded p-values are statistically significant (<0.002). β_1 refers to the estimate of RHI association with ODI slope in Equation 1.

Regions	12 month RHI *count* estimate β_1	12 month RHI *count* estimate p-value	12 Month RHI *rank* estimate β_1	12 Month RHI *rank* estimate p-value
**cingulate**	1.46E-07	0.58	1.12E-05	0.08
**frontal**	1.45E-07	0.66	7.42E-06	0.36
**occipital**	2.82E-07	0.38	1.34E-05	0.08
**orbitofrontal**	-3.6E-07	0.18	-6.1E-07	0.92
**parietal**	7.84E-08	0.78	9.51E-06	0.17
**temporal**	-1.6E-07	0.42	1.96E-06	0.70

© 2025 Song JY et al. *JAMA Network Open*.

eTable 6. Linear Model Results for AD Slope

Linear models were fit to both 12-month RHI count and 12-month RHI rank. β_1 refers to the estimate of RHI association with AD slope in Equation 1.

Regions	12 month RHI *count* estimate β_1	12 month RHI *count* estimate p-value	12 Month RHI *rank* estimate β_1	12 Month RHI *rank* estimate p-value
**cingulate**	3.16E-10	0.63	-7.85E-11	>0.99
**frontal**	4.43E-10	0.46	1.43E-08	0.30
**occipital**	-9.3E-10	0.13	-3.3E-08	0.02
**orbitofrontal**	6.71E-10	0.22	1.63E-09	0.90
**parietal**	4.92E-10	0.32	1.5E-08	0.19
**temporal**	8.73E-10	0.05	1.49E-08	0.15

© 2025 Song JY et al. *JAMA Network Open*.

eTable 7. Additional Study Demographic Characteristics
Healthy amateur soccer players in the greater NYC area. Prior 12 month RHI exposure was determined via HeadCount. Information is self-reported.

Characteristic	RHI Exposure Quartile 1 (rank 8-88)	RHI Exposure Quartile 2 (rank 89.5-176)	RHI Exposure Quartile 3 (rank 177-264)	RHI Exposure Quartile 4 (rank 265.5-352)
**Average # of Alcoholic Drinks per Week (mean± std)**	2.4±0.9	2.3±0.9	2.0±0.9	2.0±0.9
**Max # of Alcoholic Drinks on One Occasion (mean± std)**	2.9±1.4	2.8±1.5	2.4±1.8	2.8±2.0
**Heart Disease Self Reported (# of participants)**	7	3	1	3
**Diabetes Self Reported (# of participants)**	8	2	2	7
**High Blood Pressure Self Reported (# of participants)**	8	5	5	6
**Stroke Self Reported (# of participants)**	1	0	3	1
**Anxiety Score (higher score, higher anxiety)**	53.70±5.22	52.13±5.77	51.47±7.51	52.30±6.19
**Depression Score (higher score, higher anxiety)**	48.36±4.93	47.84±5.34	47.83±6.94	47.47±5.87

© 2025 Song JY et al. *JAMA Network Open*.

eTable 8. Post-Hoc Analysis: Linear Model Results for Orbitofrontal FA Slope Association RHI With Additional Covariate Effect Estimates

Linear models were fit to 12-month RHI count as described in Equation 1.

**Term**	**Effect estimate**	**P-value**
(Intercept)	-0.1513126	<0.0001
Age	0.0005965	<0.0001
Biological Sex (Male)	0.00261927	0.07075064
RHI count	0.00000107	0.00050857
Concussion (1)	-0.0011846	0.50611681
Concussion (2+)	-0.0012489	0.46165903
Hx_HeartDisease (binary)	-0.0051966	0.1503551
Hx_Diabetes (binary)	-0.0025557	0.42511749
Hx_High Blood Pressure (binary)	0.00555935	0.05850567
Hx_Stroke (binary)	-0.0068992	0.20937489
AlcWeek (#)	-0.0019613	0.04656242
AlcMax (#)	-0.0002945	0.55244742
SchoolYears (#)	0.0000355	0.91236046
Anxiety Score	0.0000665	0.61002137
Depression Score	0.0000568	0.6850696

© 2025 Song JY et al. *JAMA Network Open*.
