# Supplementary material for: Orbitofrontal Gray-White Interface Injury and the Association of Soccer Heading With Verbal Learning
Source: JAMA Netw Open. 2025 Sep 18;8(9):e2532461. doi: 10.1001/jamanetworkopen.2025.32461 (PMC12447236; doi:10.1001/jamanetworkopen.2025.32461)
Supplement: Supplement 2. — Data Sharing Statement [file jamanetwopen-e2532461-s002.pdf]

Data Sharing Statement

Song. Orbitofrontal Gray-White Interface Injury and the Association of Soccer Heading With Verbal Learning. *JAMA Netw Open*. Published September 18, 2025.
doi:10.1001/jamanetworkopen.2025.32461

Data
Data available: No

Additional Information
Explanation for why data not available: Data can be shared at request
